# Influence of Different Strain Hardening Models on the Behavior of Materials in the Elastic–Plastic Regime under Cyclic Loading

**DOI:** 10.3390/ma13235323

**Published:** 2020-11-24

**Authors:** Peter Sivák, Peter Frankovský, Ingrid Delyová, Jozef Bocko, Ján Kostka, Barbara Schürger

**Affiliations:** Department of Applied Mechanics and Mechanical Engineering, Faculty of Mechanical Engineering, Technical University of Košice, Letná 9, 042 00 Košice, Slovakia; peter.sivak@tuke.sk (P.S.); ingrid.delyova@tuke.sk (I.D.); jozef.bocko@tuke.sk (J.B.); jan.kostka@tuke.sk (J.K.); barbara.schurger@tuke.sk (B.S.)

**Keywords:** material models, plastic deformation, deformation hardening, simulation

## Abstract

In exact analyses of bodies in the elastic–plastic regime, the behavior of the material above critical stress values plays a key role. In addition, under cyclic stress, important phenomena to be taken into account are the various types of hardening and the design of the material or structure. In this process, it is important to define several groups of characteristics. These include, for instance, the initial area of plasticity or load which defines the interface between elastic and plastic deformation area. The characteristics also include the relevant law of plastic deformation which specifies the velocity direction of plastic deformation during plastic deformation. In the hardening condition, it is also important to determine the position, size and shape of the subsequent loading area. The elasto-plastic theory was used for the analysis of special compliant mechanisms that are applied for positioning of extremely precise members of the Compact Linear Collider (CLIC), e.g., cryomagnets, laser equipment, etc. Different types of deformation hardening were used to simulate the behavior of particular structural elements in the elastic–plastic regime. Obtained values of stresses and deformations may be used in further practical applications or as default values in other strain hardening model simulations.

## 1. Introduction

Plastic deformation of a structural element is an irreversible change in the shape and dimensions of the body due to external forces. The stress state in which the body is permanently deformed is the state of elastic–plastic stress [1].

In practical engineering, it is generally inadmissible to create plastic deformation, plastic deformation of a critical size or plastic stability failure. Under uniaxial stress, i.e., under tension, compression or bending, the plastic deformation starts significantly at the yield stress. In other stress modes and in a multiaxial state of stress, the limit state of plastic deformation is determined by a more complex procedure using different plasticity conditions [2,3].

Due to the plastic deformation of components, the geometry is permanently changed. As long as there is a functional relation between these components, their partial or complete malfunction or the overall load-bearing loss might or might not occur [4,5].

However, in certain justified or specific cases, it is possible to consider or allow plastic deformation to occur in the whole structure or in its individual elements. The possibility of allowing plastic deformation in statically certain and especially statically indeterminate tasks (structures, systems) can significantly increase the load-bearing capacity of the structure, in some cases even by several tens of percent.

The knowledge of the materials’ behavior in elastic–plastic regime plays a fundamental role in the analysis, particularly in the case of cyclic stresses where the mutual dependence of stress and deformation is formed by a hysteresis loop. Another phenomenon, which complicates the analyses, is the behavior of structural elements when, after reaching the ultimate limit of plastic load-bearing and relieving the element, repeated loading follows [4]. If the repetitive load is in its original orientation, there is an apparent increase in the yield point, the so-called Bauschinger effect or the phenomenon of deformation hardening.

Many authors are currently dealing with the issue of assessment of structural elements in the elastic–plastic regime, considering some form of deformation hardening of classical or modern materials. A few works can be mentioned, e.g., [5,6,7,8,9,10], in which the authors deal with the influence of isotropic and kinematic reinforced material in the process of sheet metal forming using simulation methods. The influence of kinematic hardening on the properties of sheets during their drawing is discussed, for instance in [6,7]. Propagation of cracks in such strengthened materials is described, for instance in [8,9,10]. Isotropic and kinematic hardening of modern, e.g., composite materials are the topic of, for instance, [11].

In engineering practice, when designing and simulating structural elements loaded under elastic–plastic deformation conditions, a designer must also rely on methodologies and algorithms in the form offered by commercial software systems for the numerical analysis using finite element methods (FEM).

One of the basic analyses is simulating the specific properties and behavior of materials in the elastic–plastic regime under cyclic loading to capture the Bauschinger effect and strengthen the materials by it. The basic mechanisms of hardening used in numerical analyses and simulations include isotropic, kinematic, combined isotropic–kinematic and others. The effect of applying different mechanisms of deformation hardening can be obtained by the comparison of the results of stress and strain analyses of mutually identical or similar tasks (practice).

**Figure 1:**
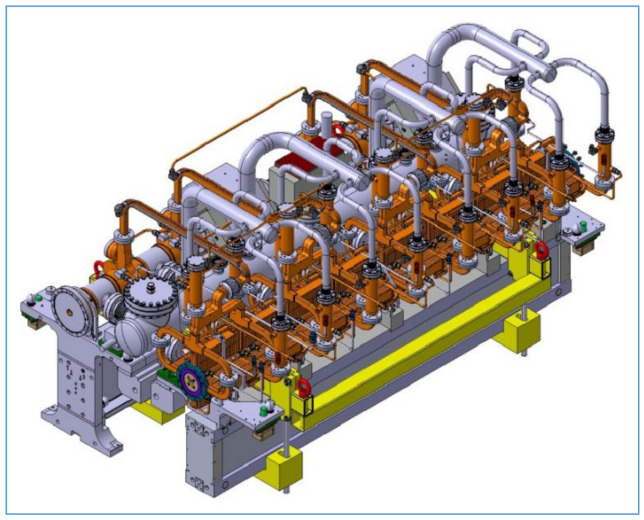
Detail of the collider assembly [12].

The analyzed structures are used for very precise placement of cryomagnets and other devices in the Compact Linear Collider (CLIC) in CERN, Figure 1. They are part of approximately 4 m long beams carrying a mass of several hundred kilograms (up to 500 kg). Altogether, several thousand positioning systems with high positioning accuracy are mounted on a length of 2 × 21 km.

Each such positioning system consists of three actuators and other parts of the positioning system. For the active alignment itself, it is necessary to pre-align the positioning systems with an accuracy of 14 µm at a distance of 200 m, relative to a straight line. To meet these strict tolerances, active alignment must be used, where sensors from the alignment systems and control actuators adjust these components to their theoretical position. The minimum effective displacement during adjustment (resolution) is less than 0.5 µm, and the repeatability of the displacement must be less than 1 µm. Due to the above-mentioned facts, a statically indeterminate system forming a special compliance mechanism was used for positioning. The subject of the present work was to analyze the deformation and strength properties of three members of this mechanism.

The components are arranged on the beams. Their external reference surfaces are mounted on V-shaped supports. Their central axis (corresponding to the theoretical axis of movement of the particle beam) is included in a cylinder with a radius of 5 µm for each beam.

**Figure 2:**
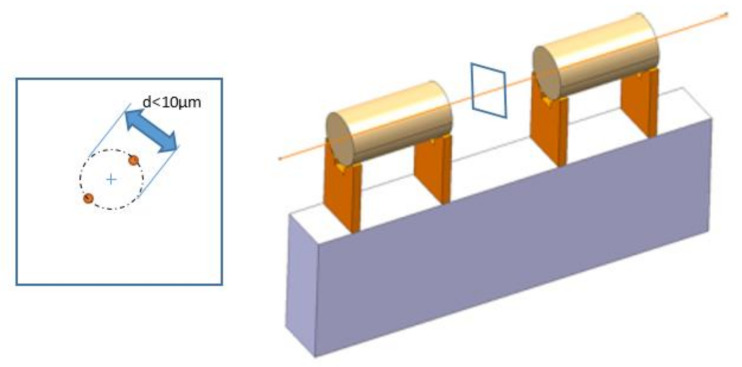
Scheme of position adjusting.

The beams are connected by a joint point geometrically defined as the intersection of the central axis of the V-shaped support of two adjacent beams in the median vertical plane. The distances between the two intersections in this plane should remain less than 10 µm, Figure 2.

## 2. An Analytical Description of Elastic–Plastic Material Behavior

An analytical description of the mechanical behavior is necessary in the analysis of structural elements made of elastic–plastic material with reinforcement [13,14,15,16,17,18]. In particular, it is necessary to define:

1. Initial area of plasticity, which specifies the interface between the elastic and plastic deformation area.
2. Law of plastic transformation, which specifies the direction of plastic deformation velocity during plastic deformation (transformation).
3. Hardening condition, which specifies the position, size and shape of the subsequent load area.

### 2.1. Initial Area of Plasticity

For the three-dimensional stress state, the criterion characterizing the transition of a material from the elastic to the plastic state can be described by the condition of plasticity, expressed by the function [15,19,20]:

(1) $$f\left(\sigma ,k_{i}\right)=0$$

where:

(2) $$\sigma ={\left[\sigma_{x},\sigma_{y},\sigma_{z},\tau_{xy},\tau_{xz},\tau_{yz}\right]}^{T}$$

is the stress vector with three normal and three shear stress components and $k_{i}\;$ are constants sufficiently describing properties of the material.

The stress vector can be generally also determined by the three principal stresses $\sigma_{1},\sigma_{2},\sigma_{3}$ and the corresponding three direction angles. When considering the isotropic material in both the elastic and the plastic region, these stresses do not depend on the choice of coordinate axes x,y,z. In such a case, the number of stress vector variables can be reduced to three by using stress invariants $I_{1},I_{2},I_{3}$ [13].

The plasticity function can be then written in the form:

(3) $$f\left(I_{1},I_{2},I_{3},k_{i}\right)\;=\;0$$

In the spatial Cartesian coordinate system described by the principal normal stresses $\sigma_{1},\sigma_{2},\sigma_{3},\;$ the plasticity condition can be represented graphically by the so-called flat plasticity [16]. The plasticity area divides the stress space into two areas. For the inner area, which is the area of elastic deformations, the following applies: f<0. For the outer area, which is the area of plastic deformations, the following applies: f>0. For the area of plasticity itself, the interface between elastic and plastic regions, the following applies f = 0.

If this area is fixed, i.e., unchanged relative to the coordinate system, and there is no change in the stresses in space when loading, unloading and reloading, this area is referred to as the initial surface of plasticity [21,22,23].

When considering a material whose plastic state is not influenced by the mean normal stress:

(4) $$\sigma_{m}=\;\left(\sigma_{1}+\sigma_{2}+\sigma_{3}\right)/3$$

The plasticity function can be expressed as a dependent variable on the components of deviatoric stress in the following form:

(5) $$f\left(s_{x},s_{y},s_{z},\tau_{xy},\tau_{xz},\tau_{yz},k_{i}\right)\;=\;0$$

Assuming isotropy of the material, this function is then expressed using the principal values of deviatoric stress:

(6) $$\left(s_{1},s_{2},s_{3},k_{i}\right)\equiv f\left(\sigma_{1}-\sigma_{m},\sigma_{2}-\sigma_{m},\sigma_{3}-\sigma_{m},k_{i}\right)=0$$

**Figure 3:**
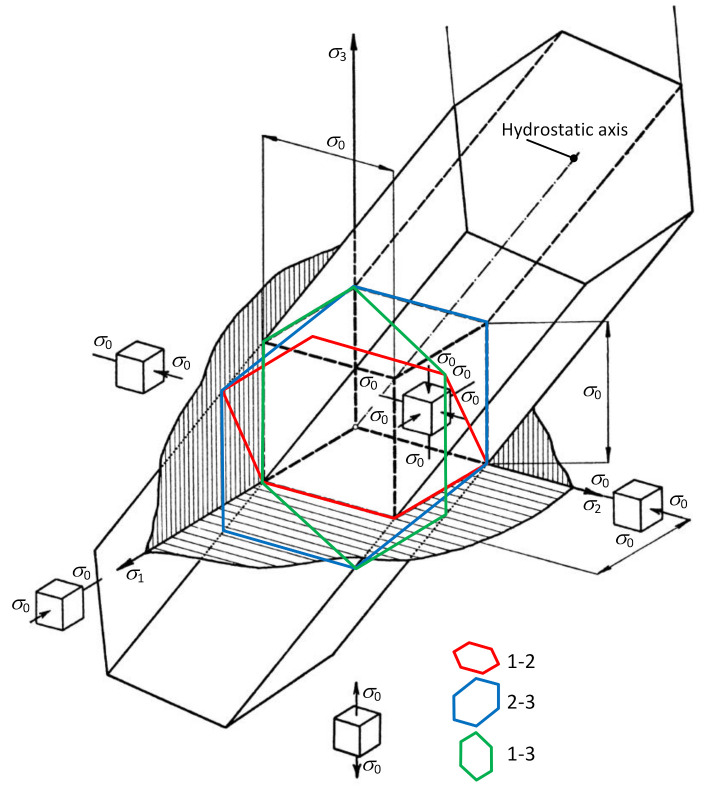
Limit prism of plasticity.

The particular geometric shape of the plasticity area in the three-dimensional space of principal stresses depends on the theory applied to the plasticity condition. In the case of shear stress stability (von Mises), the plasticity condition is expressed by the limit cylindrical area of plasticity. In the case of maximum shear stress stability (Tresca), the plasticity condition is expressed by the boundary area of the regular hexagonal prism of plasticity (see Figure 3). $\sigma_{0}$ is the relevant critical stress (yield strength of the material). Three spatial limit hexagons of plasticity 1-2, 2-3 and 1-3 as the intersections of the limit prism of plasticity and three coordinate planes are also marked in Figure 3. The axis of the respective area is identical to the so-called hydrostatic axis which passes through the origin of the coordinate system and is equidistant from all three major axes [24,25,26,27,28,29].

### 2.2. Law of Plastic Transformation

The law of plastic deformation is closely related to the condition of plasticity on the basis of the so-called plastic potential theory. The relevant theory which applies to strengthened materials can be formulated in terms of convexity and normality [30,31,32,33,34].

Depending on the convexity condition, the loading or plasticity area decomposes on one side of its tangent plane and is therefore convex. Depending on the normality condition at the regular points of the loading area, the plastic deformation velocity vector ${\dot{\vec{\varepsilon }}}^{p}$ is normal to the loading area [13,35,36,37,38,39].

The law of plastic transformation is an associative law directly formulated from the condition of normality. It assumes functional identity of loading areas with the function of plastic potential. If vectors ${\dot{\varepsilon }}^{p}$ and $F\equiv \left(\partial f/\partial \sigma \right)$ act in the direction of the outer normal to the loading area and at the end point of the vector σ, i.e., at the loading point, then both vectors act on the same beam and the rate of plastic deformation is expressed in the form [13]:

(7) $${\dot{\varepsilon }}^{p}=F\dot{\lambda }\equiv \frac{\partial f}{\partial \sigma }\dot{\lambda }$$

The parameter $\dot{\lambda }$ is a proportionality factor that is always non-negative and of indeterminate size. Therefore, vector ${\dot{\varepsilon }}^{p}$ is also of indeterminate size.

The last relation shows that in the incremental theory of plasticity, the rate (increment) of plastic deformation ${\dot{\varepsilon }}^{p}$ is expressed as a function of instantaneous stress, strain rate and stress rate [40,41,42,43,44,45]:

(8) $${\dot{\varepsilon }}^{p}={\dot{\varepsilon }}^{p}\left(\sigma ,\dot{\varepsilon },\dot{\sigma }\right)$$

**Figure 4:**
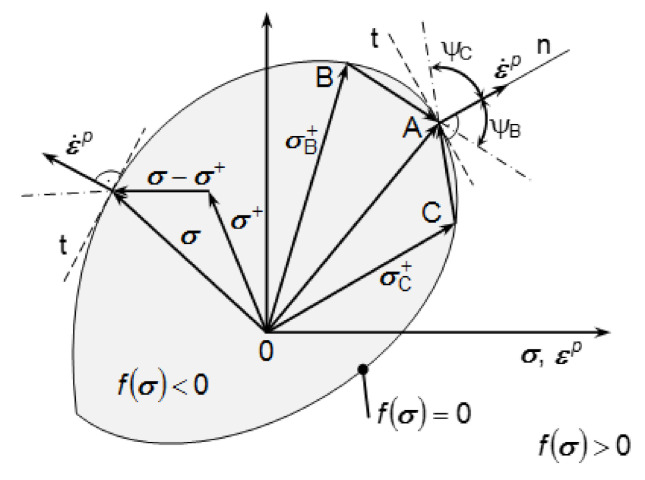
Convexity of the loading area shown on its planar section.

The plastic potential theory is equivalent to the principle of maximum dissipation rate. According to this principle, the actual dissipation rate $\sigma^{T}{\dot{\varepsilon }}^{p}$ corresponding to a given plastic deformation rate ${\dot{\varepsilon }}^{p}\;$ is greater than the fictive dissipation rate ${\left(\sigma^{+}\right)}^{T}\;{\dot{\varepsilon }}^{p}$, which is expressed from the actual plastic deformation rate ${\dot{\varepsilon }}^{p}$ and a certain stress $\sigma^{+}$ on or within the surface (Figure 4). Thus, the principle of maximum dissipation rate implies [46,47,48,49]:

(9) $${\left(\sigma -\sigma^{+}\right)}^{T}\;{\dot{\varepsilon }}^{p}\geq 0$$

The sign of equality applies only in the special case of neutral loading.

The scalar product of vectors ${\left(\sigma -\sigma^{+}\right)}^{T}$ and ${\dot{\varepsilon }}^{p}$ defines a blunt, i.e., a right or an acute angle between these vectors for any $\sigma^{+}$. At the same time, the plastic deformation velocity vector ${\dot{\varepsilon }}^{p}$ must be normal to the loading area and the loading area itself must be convex [50,51,52,53,54].

### 2.3. Condition and Function of Hardening

The reinforcement condition, specifying the position, size and shape of the subsequent loading area, is determined by the dependence on the plastic history π which corresponds to $\varepsilon^{p}\;$ and $k_{i}$. History parameters can be expressed in simpler cases either by dissipation of plastic energy ${\dot{W}}^{pl}=\sigma^{T}\;{\dot{\varepsilon }}^{p}$ according to [13]:

(10) $$\varkappa \equiv \int {\dot{W}}^{pl}\;dt=\int \sigma^{T}\;{\dot{\varepsilon }}^{p}\;dt$$

or by an equivalent plastic deformation that is proportional to the second invariant of the plastic deformation velocity deviator $J_{2}\;\left[dev\left({\dot{s}}^{p}\right)\right]\;$ according to [13]:

(11) $$\varkappa \equiv \frac{2}{\sqrt{3}}\int {\left\{J_{2}\;\left[dev\left({\dot{s}}^{p}\right)\right]\right\}}^{1/2}dt$$

The symbol $dev\left(\cdot \right)$ represents deviator. 

The hardening condition then defines the hardening function $H\left(\sigma ,\pi \right)$ which determines the subsequent loading area during plastic deformation. The hardening function is expressed as [13,55]:

(12) $$H\left(\sigma ,\pi \right)=-\frac{1}{\dot{\lambda }}\left[{\left(\frac{\partial f}{\partial \varepsilon^{p}}\right)}^{T}{\dot{\varepsilon }}^{p}+\frac{\partial f}{\partial \kappa_{i}}{\dot{\kappa }}_{i}\right]$$

The full time differential of the loading function is expressed by the relation [13,14]:

(13) $$\dot{f}=F^{T}\dot{\sigma }+{\left(\frac{\partial f}{\partial \varepsilon^{p}}\right)}^{T}\;{\dot{\varepsilon }}^{p}+\frac{\partial f}{\partial \kappa_{i}}{\dot{\kappa }}_{i}$$

or using Equation (12) according to [13]:

(14) $$\dot{f}=F^{T}\dot{\sigma }-H\dot{\lambda }$$

Components of the deformation vector ε or the deformation rate occurring in the elastic–plastic body can be additively decomposed into the elastic portion $\varepsilon^{e}$ and the plastic portion $\varepsilon^{p}$. Then, the following applies [13]:

(15) $$\varepsilon =\varepsilon^{e}+\varepsilon^{p},\;\mathrm{or}\;\dot{\varepsilon }={\dot{\varepsilon }}^{e}+{\dot{\varepsilon }}^{p}$$

The elastic components of the deformation vector $\varepsilon^{e}$ are coupled to stress vector components σ and the flexibility matrix D by the linear Hooke’s law according to [13]:

(16) $$\varepsilon^{e}=D^{-1}\sigma \;\mathrm{or}\;\;{\dot{\varepsilon }}^{e}=D^{-1}\dot{\sigma }$$

At the same time, when using Equation (7) [13]:

(17) $$\dot{\varepsilon }={\dot{\varepsilon }}^{e}+{\dot{\varepsilon }}^{p}=D^{-1}\dot{\sigma }+F\dot{\lambda }$$

Using Equation (14), the following applies [13]:

(18) $$\dot{\varepsilon }=\left(D^{-1}+H^{-1}FF^{T}\right)\;\dot{\sigma }$$

From the relation Equation (17) dependencies (relationships) can be expressed [13,14]:

(19) $$D\;\dot{\varepsilon }=\dot{\sigma }+DF\dot{\lambda }$$

and

(20) $$F^{T}D\;\dot{\varepsilon }=H\dot{\lambda }+F^{T}DF\dot{\lambda }$$

From the last relation, it is possible to express the proportionality factor [13]:

(21) $$\dot{\lambda }=\frac{F^{T}D\dot{\varepsilon }}{H+F^{T}DF}$$

Using Equations (19) and (21) and the elastic–plastic symmetrical matrix $D^{ep}$, the stress velocity vector is expressed [13]:

(22) $$\dot{\sigma }=\left[D-\frac{DFF^{T}D\;}{H+F^{T}DF}\right]\;\dot{\varepsilon }=D^{ep}\dot{\varepsilon }$$

The previous relationships determine the general reinforcement condition and the function of hardening which determines the subsequent loading area during plastic deformation. They can be further modified for specific cases to accurately describe individual modifications of material models, e.g., a thermoplastic or elastic–plastic material, either with or without deformation hardening, etc. [56,57,58,59,60]

### 2.4. Isotropic Hardening 

**Figure 5:**
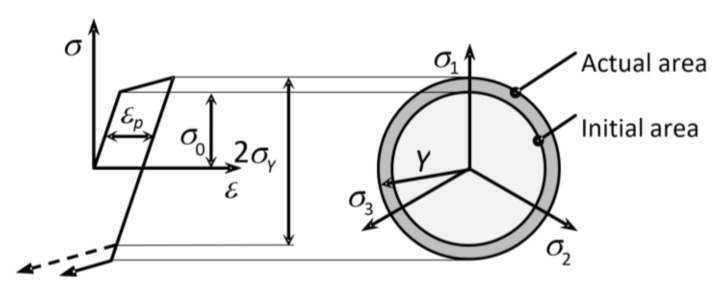
Loading areas for an isotropically hardened material.

In the case of isotropic hardening, the loading or plasticity area increases in all directions equally and it is geometrically similar to the initial area. For isotropic hardening, e.g., in the case of the von Mises plasticity condition, only the radius of the plasticity area changes (see Figure 5). Generally, for the isotropic state of the loading area, Equation (23) applies [13]:

(23) $$f\left(\sigma ,Y\right)=\bar{f}\left(\sigma \right)-Y=0$$

where Y is a scalar variable related to the development of dislocation structures during loading.

The material model with pure isotropic hardening can be used with sufficient accuracy only in the case of monotonic loading in technological operations such as forming. In the case of cyclic loading, this principle of hardening is not sufficiently precise, as it fails to describe the Bauschinger effect (see Figure 5). It also does not correspond to the typical behavior of ductile materials [13]:

### 2.5. Kinematic Hardening

**Figure 6:**
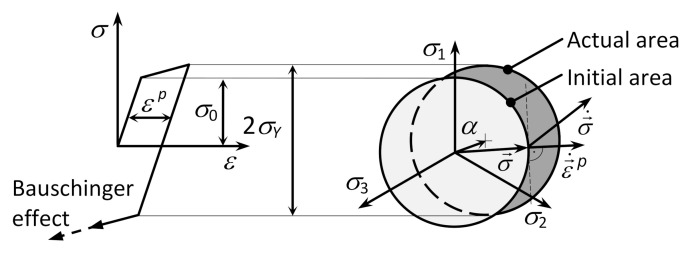
Loading areas for a kinematically hardened material.

Kinematic hardening is applied in the cases where the center of the loading or plasticity area is displaced non-symmetrically with respect to the origin of the coordinate system. The size and the shape of the area do not change at all in this case but move as a rigid unit beyond the loading point (see Figure 6) [13].

The internal control variable is the kinematic stress tensor *α* which defines the actual position of the center of the plasticity area and it is related to the internal stress. Then, in purely kinematic hardening, the plasticity condition is expressed in the form of [36,37,38,39,40,41,42,43,44,45]:

(24) $$f\left(\sigma ,\alpha \right)=\bar{f}\left(\sigma -\alpha \right)-\sigma_{Y}=0$$

Purely kinematic hardening can be used for steel whose yield strength is similar to both the static and cyclic deformation curve [35,36,37,38,39].

## 3. Numerical Simulations of the Effects of Isotropic and Kinematic Hardening

A series of analyses were performed in the SolidWorks programming environment (2012; Dassault Systèmes SolidWorks Corporation, Waltham, MA, USA) to verify the degree of consistency of the simulation results of different methods of material hardening. These analyses were based on the methodology of applying different material models in the conditions of uniaxial or multiaxial stress for basic stress modes [61,62,63,64]. 

**Figure 7:**
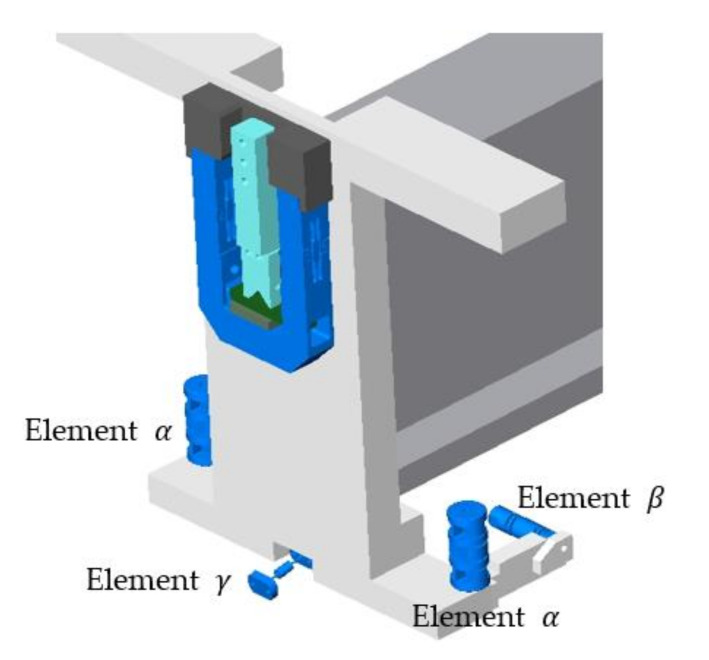
Detail of the location of the elastic members in the positioning frame.

**Figure 8:**
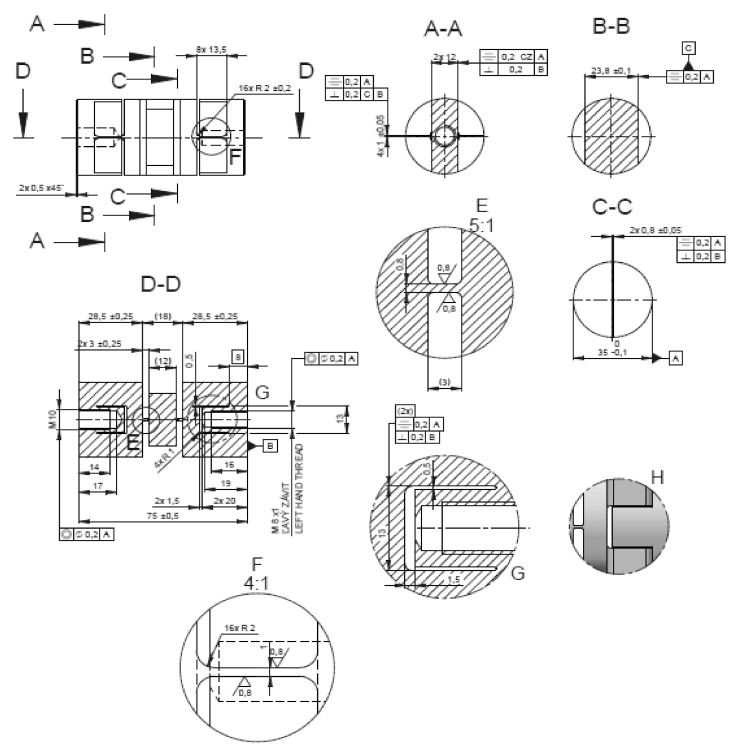
Shape and dimensions of the element *α*.

**Figure 9:**
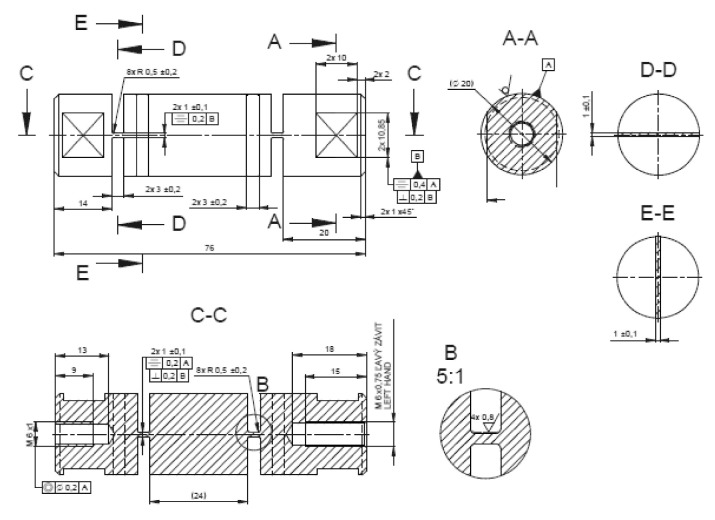
Shape and dimensions of the element *β*.

**Figure 10:**
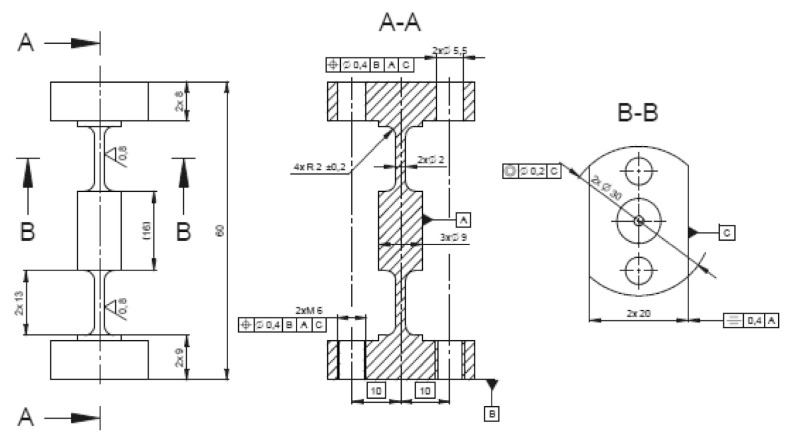
Shape and dimensions of the element *γ*.

Virtual objects of these analyses were a group of structural elements—universal joints in the form of elastic elements, internally designated as *α*, *β*, *γ*, Figure 7, Figure 8, Figure 9 and Figure 10. Real models of these elements were developed by ZTS VVÚ Košice (Košice, Slovakia) and they are part of a positioning device on the Compact Linear Collider of the European Organization for Nuclear Research (CERN).

Two methods of virtual loading were applied, identical to the assumed stress of real elements using the isotropic and kinematic model of material hardening. The first one included tension above the yield strength followed by relief and the second one included torsion above the yield strength followed by relief. The torsion was done in both directions.

The following study parameters were used:

- control method: controlled by force increments
- iterative method: Newton-Raphson method
- integration method: Newmark method.

Individual simulations were done using isotropic and kinematic models of material strengthening.

In this paper, the elements *α* and *β* were chosen for a non-linear static study in the FEM programming environment. The results of some computations were compared.

The elastic members of the positioning system with extremely high positioning accuracy were subjected to numerical simulation. Such positioning systems are intended for positioning cryomagnets, laser devices and other special technologies and devices used in particle accelerators used in particle physics experiments. The solution of the positioning system for positioning compact linear cryomagnets is adapted to the new original concept of two-beam acceleration of the accelerator itself, in which high-frequency power is obtained from a low-energy particle beam, which has a high intensity and is transmitted to a parallel high-energy accelerating beam.

For the individual basic parts of the positioning system, such as the supporting members, the actuating members of the joint, their conceptual structural solutions were designed. The positioning frame includes elastic members, Figure 7, Figure 8, Figure 9 and Figure 10. To verify the required strength and stiffness of the proposed parts, as well as of the whole support system for positioning the cryomagnets, control computations were realized by the finite element method.

Numerical analyses included monitoring of differences or, on the contrary, the degree of conformity of relevant simulation results, which included information on the distribution and magnitude of the maximum equivalent stresses and deformations, the course of mutual dependence of stresses and deformations for the individual hardening models used. The plasticity condition—shear stress intensity constancy (von Mises)—was chosen according to the conclusions of the analyses described in articles [45,46,47,48,49,50,51,52,53].

### 3.1. Numerical Simulation with Tensile Stress Followed by Unloading

**Figure 11:**
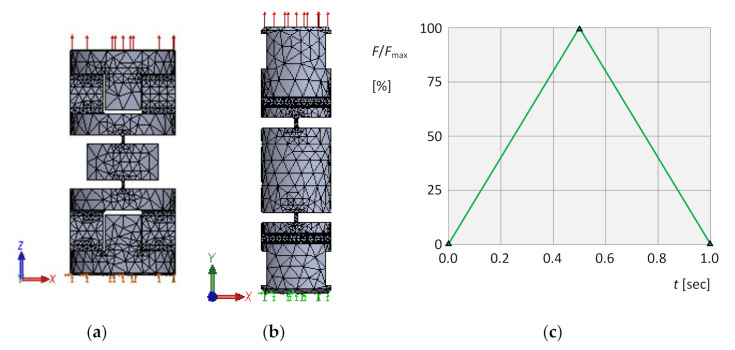
Tensile simulations. (**a**) Meshing, boundary conditions and load for the element *α*. (**b**) Meshing, boundary conditions and load for the element *β*. (**c**) Time dependence of the loading force.

The elements are made of material 34CrNiMo6 with basic material properties: the Young’s modulus $2.1\cdot 10^{6}\;\mathrm{MPa}$, the Poisson’s ratio 0.3, the yield stress 900 MPa, the tensile strength 1100 MPa. For this group of simulations, meshing, boundary conditions and load of the virtual object were chosen (Figure 11a,b) with the time-dependent chart of loading according to Figure 11c. In all computations, the size of elements in critical points (e.g., notches) was approximately 0.1 mm. For meshing, parabolic tetrahedral solid elements were used.

This virtual object was then subjected to a numerical simulation using an isotropic and kinematic hardening model. In this way, fields and extremes of stresses and deformations were obtained.

**Figure 12:**
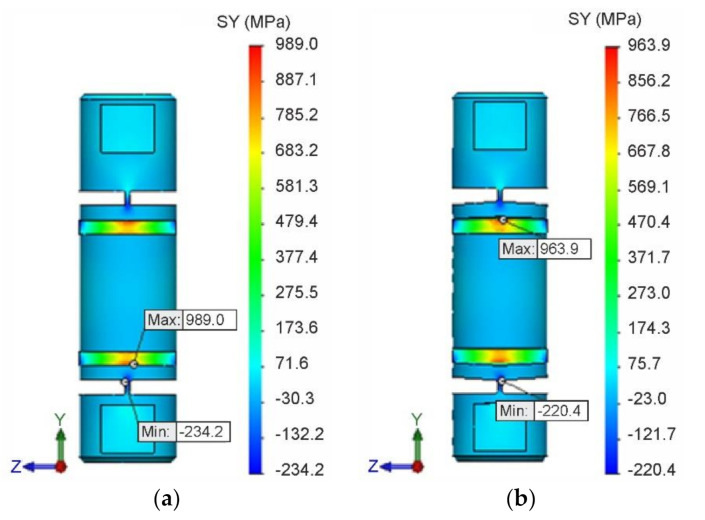
Field and extreme values of normal stresses. (**a**) $\sigma_{\mathrm{yI}}\;$ for isotropic hardening. (**b**) $\sigma_{\mathrm{yK}}$ for kinematic hardening.

The field of normal stresses $\sigma_{\mathrm{yI}}$ in the longitudinal *y*-axis direction for isotropic hardening is shown in Figure 12a, and the stress field $\sigma_{\mathrm{yK}}$ for kinematic hardening is shown in Figure 12b. At the same time, the locations and magnitudes of extreme values $\sigma_{\mathrm{yI}\max}$, $\sigma_{\mathrm{yI}\min}$, $\sigma_{\mathrm{yK}\max}$ and $\sigma_{\mathrm{yK}\min}$ of these stresses are identified.

**Figure 13:**
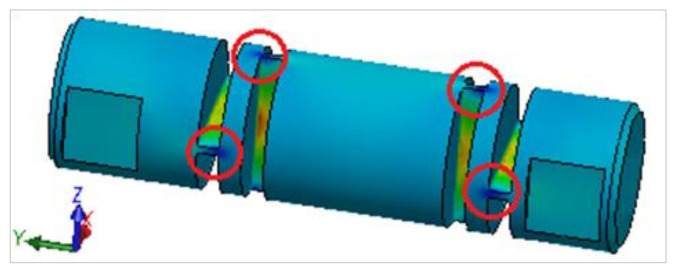
Identification of concentration points of compressive stresses using the isotropic material hardening model.

Another interesting feature is the occurrence of compressive normal stresses. These probably occurred due to complex shape configurations and stress concentrators. Figure 13 identifies stress concentration points of these compressive stresses using the isotropic material hardening model.

**Figure 14:**
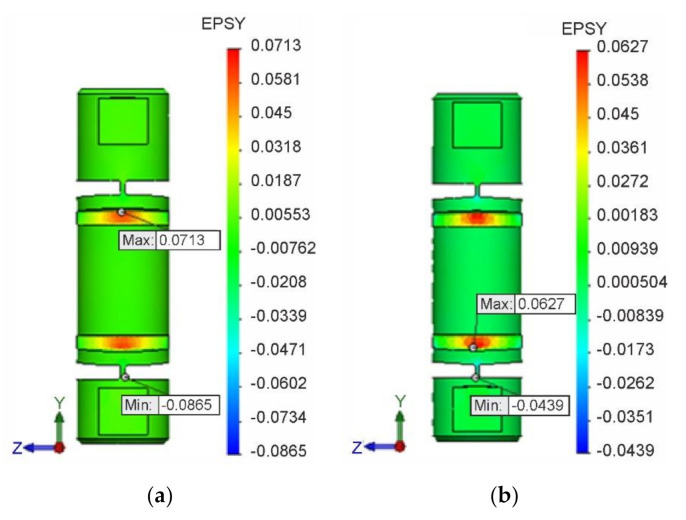
Field and extreme values of strains. (**a**) $\varepsilon_{\mathrm{yI}}\;$ for isotropic hardening. (**b**) $\varepsilon_{\mathrm{yK}}$ for kinematic hardening

The field of strains $\varepsilon_{\mathrm{yI}}$ in the longitudinal y-axis direction for isotropic hardening is shown in Figure 14a, and the field of strains $\varepsilon_{\mathrm{yK}}$ for kinematic hardening in Figure 14b. At the same time, the locations and magnitudes of extreme values $\varepsilon_{\mathrm{yI}\max}$, $\varepsilon_{\mathrm{yI}\min}$, $\varepsilon_{\mathrm{yK}\max}$ and $\varepsilon_{\mathrm{yK}\min}$ of these deformations are identified.

**Figure 15:**
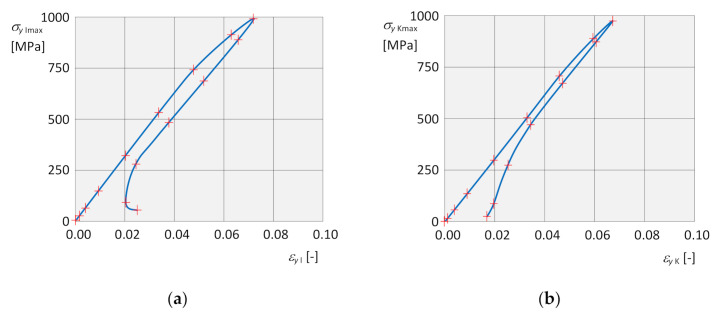
Graphical dependencies. (**a**) of stress $\sigma_{\mathrm{yI}\max}$ on deformation $\varepsilon_{\mathrm{yI}}$ for isotropic hardening; (**b**) of stress $\sigma_{\mathrm{yK}\max}$ on deformation $\varepsilon_{\mathrm{yK}}$ for kinematic hardening.

The dependence of the instantaneous maximum normal stress $\sigma_{\mathrm{yI}\max}$ on the corresponding instantaneous strain $\varepsilon_{\mathrm{yI}}\;$ at the respective isotropic hardening point is shown in Figure 15a, and the dependence $\sigma_{\mathrm{yK}\max}$ on $\varepsilon_{\mathrm{yK}}\;$ for kinematic hardening in Figure 15b, in both cases for the entire loading cycle. In both dependencies, it is also possible to clearly identify residual normal stresses $\sigma_{\mathrm{yI}\mathrm{res}}$, $\sigma_{\mathrm{yK}\mathrm{res}}$ and residual strains $\varepsilon_{\mathrm{yI}\mathrm{res}}$, $\varepsilon_{\mathrm{yK}\mathrm{res}}$ due to plastic deformation after the end of loading.

When comparing all corresponding variables Q in the tensile simulation for both hardening models, their relational dependence $\left|Q_{\;I}\right|>\left|Q_{\;K}\right|$ was found, namely: $\left|\sigma_{\mathrm{yI}\;\max}\right|>\left|\sigma_{\mathrm{yK}\;\max}\right|$, $\left|\sigma_{\mathrm{yI}\;\min}\right|>\left|\sigma_{\mathrm{yK}\;\min}\right|$, $\left|\varepsilon_{\mathrm{yI}\;\max}\right|>\left|\varepsilon_{\mathrm{yK}\;\max}\right|$, $\left|\varepsilon_{\mathrm{yI}\;\min}\right|>\left|\varepsilon_{\mathrm{yK}\;\min}\right|$, $\left|\sigma_{\mathrm{yI}\;\mathrm{res}}\right|>\left|\sigma_{\mathrm{yK}\;\mathrm{res}}\right|$, $\left|\varepsilon_{\mathrm{yI}\;\mathrm{res}}\right|>\left|\varepsilon_{\mathrm{yK}\;\mathrm{res}}\right|$.

### 3.2. Numerical Simulation with Torsional Stress Followed by Unloading

**Figure 16:**
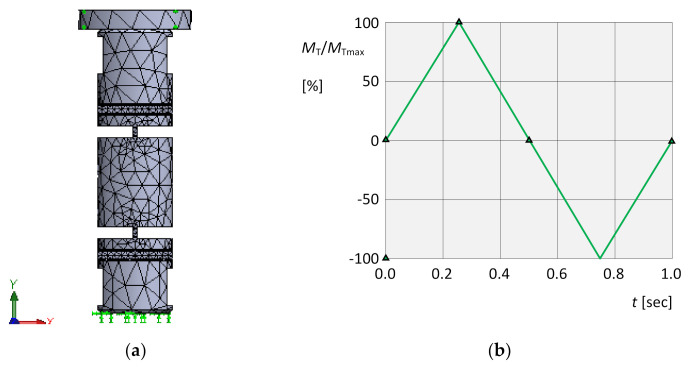
Torsional simulations. (**a**) Meshing, boundary conditions and load. (**b**) Time dependence of the loading torque.

For this group of simulations, meshing and boundary conditions of the virtual object were chosen according to Figure 16a. A controlled loading process with a loading torque acting around the *y*-axis (Figure 16a) with both orientations and time course according to Figure 16b was applied.

This virtual object was then subjected to the numerical simulation using an isotropic and kinematic hardening model. In this way, fields and extremes of stresses and deformations were obtained.

**Figure 17:**
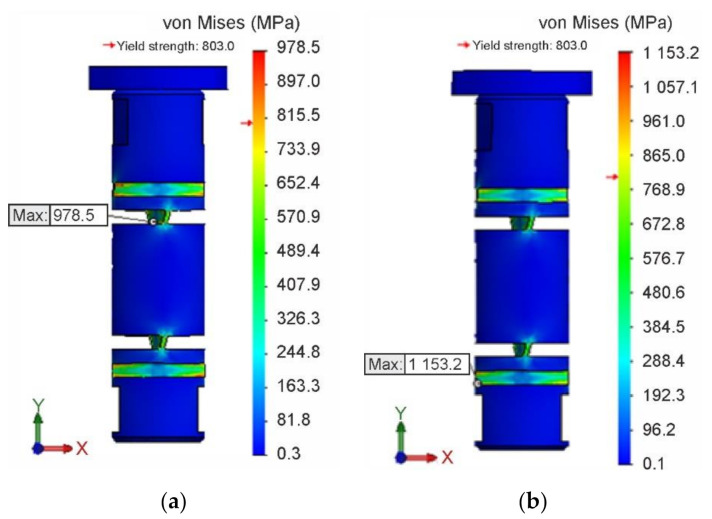
Field and extreme values of equivalent stresses. (**a**) $\sigma_{\mathrm{VM}\;I}$ for isotropic hardening. (**b**) $\sigma_{\mathrm{VM}K}$ for kinematic hardening.

The field of equivalent stresses $\sigma_{\mathrm{VM}I}$ according to von Mises theory for isotropic hardening is shown in Figure 17a, and the field of equivalent stresses $\sigma_{\mathrm{VM}K}$ for kinematic hardening in Figure 17b. At the same time, locations and magnitudes of extreme values $\sigma_{\mathrm{VM}I\max}$ and $\sigma_{\mathrm{VM}K\;\max}$ of these stresses are identified. The values $\sigma_{\mathrm{VM}I\max}$ and $\sigma_{\mathrm{VM}K\max}$ for both isotropic and kinematic hardening were achieved in the second loading cycle, i.e., in the torsion in the opposite direction. However, unlike the isotropic hardening simulation, in the latter case, the maximum values of the equivalent stresses $\sigma_{\mathrm{VM}K\max}$ exceeded the (strength) limit of the material. Simulation conditions were the same in both cases. Thus, it can be assumed that if a material model with kinematic hardening is applied, the structural element would be damaged.

**Figure 18:**
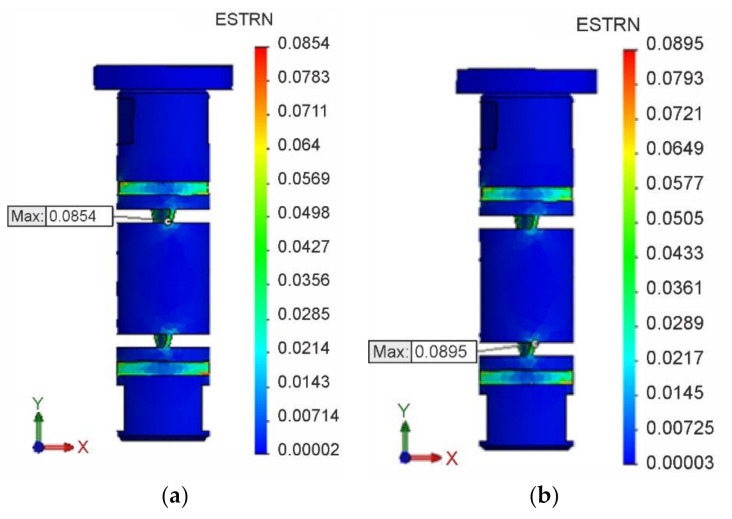
Field and extreme values of strains. (**a**) $\varepsilon_{\mathrm{eq}\;I}$ for isotropic hardening. (**b**) $\varepsilon_{\mathrm{eq}\;K}$ for kinematic hardening.

The field of equivalent von Mises strains $\varepsilon_{\mathrm{eq}I}$ for isotropic hardening is shown in Figure 18a, and the field of equivalent von Mises strains $\varepsilon_{\mathrm{eq}K}$ for kinematic hardening in Figure 18b. At the same time, the locations and magnitudes of extreme values $\varepsilon_{\mathrm{eq}I\max}$ and $\varepsilon_{\mathrm{eq}K\max}$ of these deformations are identified. 

As in the case of stress analysis, the values $\varepsilon_{\mathrm{eq}I\max}$ and $\varepsilon_{\mathrm{eq}K\max}$ for both isotropic and kinematic hardening were achieved in the second loading cycle, i.e., in the torsion in the opposite direction.

**Figure 19:**
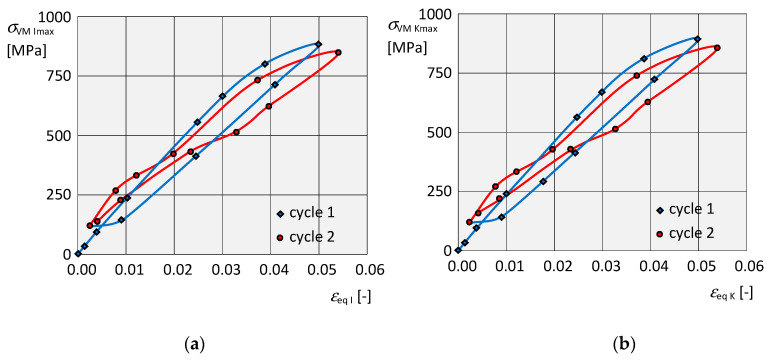

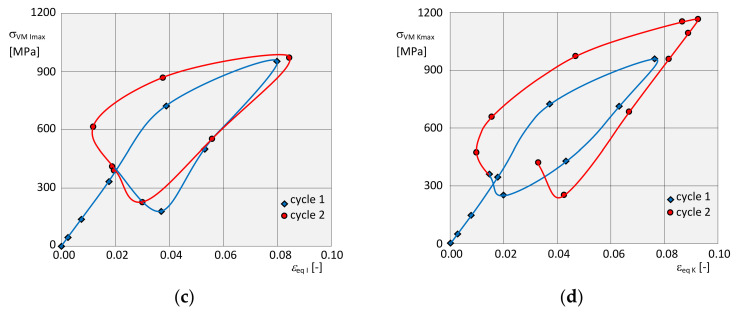
Graphical dependencies. (**a**) Of stress $\sigma_{\mathrm{VM}\;I\;\max}$ on deformation $\varepsilon_{\mathrm{eq}\;I}$ for isotropic hardening of the element *α*. (**b**) Of stress $\sigma_{\mathrm{VM}\;K\;\max}$ on the deformation $\varepsilon_{\mathrm{eq}\;K}$ for kinematic hardening of the element *α*. (**c**) Of stress $\sigma_{\mathrm{VM}\;I\;\max}$ on the deformation $\varepsilon_{\mathrm{eq}\;I}$ for isotropic hardening of the element *β*. (**d**) Of stress $\sigma_{\mathrm{VM}\;K\;\max}$ on the deformation $\varepsilon_{\mathrm{eq}\;K}$ for kinematic hardening of the element *β*.

The dependence of the instantaneous maximum equivalent stress $\sigma_{\mathrm{VM}I\max}$ on the corresponding instantaneous strain $\varepsilon_{\mathrm{eq}I}$ at the respective isotropic hardening point is shown for α,β in Figure 19a,c, and the dependence $\sigma_{\mathrm{VM}K\max}$ on $\varepsilon_{\mathrm{eq}K}$ for kinematic hardening for *α*, *β* in Figure 19b,d, in both cases for two complete loading cycles. In both dependencies, it is also possible to clearly identify residual normal stresses $\sigma_{\mathrm{VM}I\mathrm{res}}$, $\sigma_{\mathrm{VM}K\mathrm{res}}$ and residual strains $\varepsilon_{\mathrm{eq}I\mathrm{res}}$, $\varepsilon_{\mathrm{eq}K\mathrm{res}}$ due to the plastic deformation after the end of loading. In addition, at the end of each of the two load cycles, the stresses began to increase slightly again.

When comparing all corresponding variables Q in the torsional simulation for both hardening models, their relational dependence $\left|Q_{\;I}\right|<\left|Q_{\;K}\right|$ was found, which is opposite as in the tensile simulation, namely: $\left|\sigma_{\mathrm{VM}\;I\;\max}\right|<\left|\sigma_{\mathrm{VM}\;K\;\max}\right|$, $\left|\varepsilon_{\mathrm{eq}\;I\;\max}\right|<\left|\varepsilon_{\mathrm{eq}\;K\;\max}\right|$, $\left|\sigma_{\mathrm{VM}\;I\;\mathrm{res}}\right|<\left|\sigma_{\mathrm{VM}\;K\;\mathrm{res}}\right|$, $\left|\varepsilon_{\mathrm{eq}\;I\;\mathrm{res}}\right|<\left|\varepsilon_{\mathrm{eq}\;K\;\mathrm{res}}\right|$.

## 4. Conclusions

When designing a positioning system for cryomagnet positioning, all proposed parts must be fully functional. To verify the required strength and stiffness of the proposed parts of the supporting system of cryomagnet positioning, it was necessary to perform control computations and analyses using FEM. In the present article, attention was paid to the flexible members (special compliance mechanisms), which are part of the positioning device of the collider magnets at CERN. Simulations were performed for isotropic and kinematic hardening of individual elastic members. 

Based on the thorough and detailed examination and comparison of the obtained data, several preliminary partial conclusions can be stated.

In the first case, each member was stressed to pull above the yield point with consequent relief. In the second case, the members were subjected to torsional stresses equally above the yield point with consequent relief, the torsion being applied first to one side and then to the other. Differences between the maximum stresses, displacements and stress-strain curves were observed using individual hardening models. Their difference was on the order of a few percent, in extreme cases even several tens of percent, depending on the quantity considered.

For the individual elastic members, the resulting values of stresses, deformations and their dependence curves for individual types of hardenings were the same, possibly comparable. From the curves of the dependence of the stress on the deformation under torsional load for the elastic element *α*, a slight difference in the second cycle during unloading is evident, Figure 19a,b. For the elastic element *β*, it is clear from the dependence that the stresses at the end of both cycles started to rise again, Figure 19c,d. Under torsional stress, the results were specific to the individual elastic members, so it was not possible to draw general conclusions. 

The results of the calculations show that the elastic members are suitable for the given load. Moreover, the element stiffnesses were used for further analysis of structural deformations.

However, the above-stated conclusions can be considered preliminary due to the limited number of loading cases. At the same time, they were influenced by specific configurations of design and stress conditions of structural elements without any experimental verification.
